# Development of a Rapid Molecular Detection System for Green Mold Disease of *Ganoderma lingzhi* Caused by *Trichoderma longibrachiatum*

**DOI:** 10.3390/life16060964

**Published:** 2026-06-08

**Authors:** Wenlong Zhao, Chunlan Zhang, Jize Xu, Yuanju Jin

**Affiliations:** 1 College of Landscape Architecture, Changchun University, Changchun, 130000, China; 18968152586@163.com (W.Z.);; 2 Agricultural College, Jilin Agricultural Science and Technology University, Jilin 132101, China

**Keywords:** *Trichoderma longibrachiatum*, *Ganoderma lingzhi*, green mold disease, PCR detection, molecular diagnosis

## Abstract

Green mold disease caused by *Trichoderma* species represents one of the most serious threats to the cultivation of *Ganoderma lingzhi*. In this study, a rapid molecular detection system was developed for the identification of green mold caused by *Trichoderma longibrachiatum*. The pathogen was first identified based on morphological characteristics and pathogenicity tests. Species-specific primers were then designed targeting the RPB2 gene region, and their specificity and sensitivity were evaluated using polymerase chain reaction (PCR). The results confirmed that the causal pathogen was *T*. *longibrachiatum*. The designed primers exhibited high specificity and produced amplification only with genomic DNA from the target strain. Sensitivity assays demonstrated that the minimum detectable DNA concentration was 10^−2^ ng/µL. In soil inoculation experiments, the target DNA could be detected in soil containing 4.28 × 10^6^ spores/g using the developed primers. Compared with conventional culture-based detection methods, the molecular detection system established in this study is faster, more accurate, and highly sensitive. This method provides a practical diagnostic tool for the early detection and management of green mold disease in *G*. *lingzhi* cultivation.

## 1. Introduction

China is rich in *Ganoderma germplasm* resources; however, only four species have been successfully cultivated and commercialized to date: *Ganoderma lingzhi*, *G. sinense*, *G. leucocontextum*, and *G. tsugae* [1]. *Ganoderma lingzhi* Sheng H. Wu, Y. Cao & Y.C. Dai is a traditional and precious Chinese medicinal material with various physiological activities and pharmacological effects [2], with a history of over 60 years of commercial production in China. The main production areas include Sichuan, Shandong, Fujian, Jilin, and several other provinces, where soil-covered cultivation is mainly practiced [3]. In recent years, domestic market demand for *G. lingzhi* has been increasing at an annual rate of 18–30% [4]. In 2015, the cultivation area of *G. lingzhi* in China reached about 100 km^2^, and the output of *G. lingzhi* and its spore was about 120,000 tons, with a value of 1.6 billion US dollars, accounting for about 75% and 30% of the world’s total production, respectively. Thus, China has become the world’s leading producer and exporter of both fruiting bodies and spores [5].

The number of *G. lingzhi* growers has increased with the expanding market. However, this rapid expansion has been accompanied by increasingly severe outbreaks of fungal diseases, particularly green mold caused by *Trichoderma* species, which pose a serious threat to yield and quality. A wide range of *Trichoderma* species have been reported as pathogens, with the predominant pathogenic species including *Trichoderma pleuroticola* [6], *T. harzianum* [7], *T. koningiopsis* [8], *T. hengshanicum* [9], *T. atroviride* [10], *T. longibrachiatum* [11], *T. atrobrunneum* [12], and *T. ganodermatis* [13]. *Trichoderma* species represent the primary fungal pathogens in *Ganoderma lingzhi* cultivation, with their pathogenicity involving multiple mechanisms, including competition for space and nutrients with the host, as well as the production of toxic substances, extracellular enzymes, volatile organic compounds, and secondary metabolites.

These fungi are the primary pathogens in *Ganoderma lingzhi* cultivation, with their pathogenicity involving multiple mechanisms, including competition for space and nutrients with the host, as well as the production of toxic substances, extracellular enzymes, volatile organic compounds, and secondary metabolites [14]. To better understand how they cause disease, here is a closer look at the main virulence factors. *Trichoderma* species employ a diverse set of pathogenicity factors that contribute to their virulence on *Ganoderma lingzhi*. These mechanisms fall into four main categories: cell wall degradation, secretion of toxic secondary metabolites, emission of volatile organic compounds (VOCs), and enzymatic destruction of host tissues. For cell wall degradation, *Trichoderma* produces extracellular enzymes such as chitinases, β-1,3-glucanases, and proteases, which break down the structural components of fungal cell walls and help the pathogen penetrate host tissues [14]. A recent review pointed out that these enzymes give *Trichoderma* access to host resources. Regarding secondary metabolites, Trichoderma releases a wide range of antifungal compounds, including peptaibols, polyketides, terpenoids, gliotoxin, and trichokonins. For example, *T. harzianum* produces viridiofungin A, which completely inhibits spore germination of plant pathogens, while metabolites from *T. longibrachiatum* achieve about 80% disease control at 500 μg/mL. Volatile organic compounds like 6-pentyl-α-pyrone—a common *Trichoderma* metabolite—have been shown to suppress plant pathogenic microorganisms and also activate plant defense responses. Finally, *Trichoderma* also secretes various hydrolytic enzymes that disrupt host cell integrity and weaken infected tissues, thereby promoting disease progression. Taken together, these pathogenic mechanisms enable *Trichoderma* species to establish infection, outcompete the host, and cause serious damage in *G. lingzhi* cultivation.

Green mold is a significant fungal disease affecting the fruiting bodies of *Ganodermataceae* species. The disease can occur throughout the entire cultivation cycle of *G. lingzhi*. Severe infection during the mycelial growth stage may prevent the formation of fruiting bodies. Infection of the fruiting bodies typically occurs during the mid-to-late cultivation period, primarily through pores and wounds. Subsequently, a green mold layer develops on the underside of the pileus, ultimately leading to decay and shrinkage of the fruiting bodies [15].

In the early stages of infection, the gray-white mycelium of Trichoderma is difficult to distinguish from the white mycelium of healthy *G. lingzhi* colonies, delaying visual diagnosis. As the disease progresses, yield losses can be severe, sometimes resulting in complete crop failure. Moreover, under continuous cropping, Trichoderma spp. accumulate in the soil, leading to smaller, deformed fruiting bodies and slower growth [16]. Thus, the same field should avoid repeated cultivation as far as possible, and not be cultivated again within three years; otherwise, serious economic losses may occur [17].

Traditional detection methods for Trichoderma species still rely heavily on morphological identification, pathogen isolation, and physiological–biochemical characterization. Morphological identification means growing the suspected pathogen on selective media and then looking at colony appearance, hyphal branching patterns, and conidial shape under a microscope [18]. Pathogen isolation usually involves plating infected tissue samples onto artificial media and waiting several days to weeks before you can examine any colonies. Physiological and biochemical characterization adds extra tests, like carbon source utilization profiles or enzyme activity assays. Although these methods are still commonly used, they come with pretty serious drawbacks. First, they take a long time, often 5 to 14 days, to get a confirmation. That kind of delay can easily make you miss the window for timely disease control. Second, they depend heavily on the operator’s taxonomic expertise. Even specialists find it notoriously difficult to tell Trichoderma species apart just by morphology, because many species share very similar features. Third, these methods are not very sensitive. They often fail to pick up the pathogen when spore levels are low, especially during the early, symptom-free stage of infection. As a result, diagnosis usually happens only after visible symptoms show up, by which time the pathogen load is already high. All these limitations point to one thing: we urgently need faster, more reliable, and more sensitive ways to detect *T. longibrachiatum* in *G. lingzhi* cultivation.

Beyond the studies conducted in China, green mold caused by Trichoderma species has been recognized as a major threat to edible mushroom cultivation worldwide. For instance, *Trichoderma aggressivum* has caused severe economic losses in the button mushroom (*Agaricus bisporus*) industry across North America and Europe, and similar outbreaks have been reported for *Pleurotus* spp. in several countries. A recent international review emphasized that the most promising diagnostic tools for early detection of Trichoderma infestation are currently based on molecular methods, although further optimization for real-time, in-field application is still required. In line with this global trend, our study focused on developing a rapid and specific molecular detection system for *T. longibrachiatum* affecting *G. lingzhi*. The RPB2 gene, which we used as the target for primer design, has been widely recognized as an effective molecular marker for species-level identification of Trichoderma in several recent international studies. To our knowledge, however, no rapid PCR-based detection assay has been specifically developed for *T. longibrachiatum* causing green mold on *G. lingzhi*, although a closely related species, *T.* pleuroticola, has been reported in a previous Chinese study. Therefore, this study not only addresses a practical need in domestic *G. lingzhi* production but also adds to the broader international efforts in developing molecular diagnostic tools for mushroom diseases.

In China, to date, only that study by Huang (2018) [6] has been reported for *T. pleuroticola*; no such method exists for *T. longibrachiatum*. Although various molecular methods have been developed for the detection of *Trichoderma* species in agricultural systems, each has inherent limitations. Quantitative PCR (qPCR) offers high sensitivity but requires expensive equipment and reagents, limiting its application in resource-limited settings. Loop-mediated isothermal amplification (LAMP) is rapid and does not require thermal cycling, yet it is prone to cross-contamination due to its high amplification efficiency and open-tube operation. Other PCR-based assays have been reported for some *Trichoderma* species, but none specifically targets *Trichoderma longibrachiatum* causing green mold on *Ganoderma lingzhi*. Moreover, most existing methods focus on soil or plant samples from other crops, without validation in the unique cultivation environment of *G. lingzhi*. To address these gaps, this study reports the first rapid and specific PCR-based detection system for *Trichoderma longibrachiatum* causing green mold on *Ganoderma lingzhi*. Unlike previous methods, our assay is designed specifically for the *G. lingzhi* cultivation system, targeting a unique region of the RPB2 gene that distinguishes *T. longibrachiatum* from closely related *Trichoderma* species. This work fills a critical diagnostic void and provides a practical tool for early disease surveillance in *G. lingzhi* production.

## 2. Materials and Methods

### 2.1. Morphological Observations and Pathogenicity Tests

The strain XU0177 was originally isolated from a naturally diseased fruiting body of *Ganoderma lingzhi* showing typical green mold symptoms. The sample was collected from a commercial *G. lingzhi* cultivation base in Zuoja Town, Jilin City, China. Four additional *Trichoderma* strains—*Trichoderma atroviride* P. Karst., *Trichoderma koningiopsis* Samuels, Carm. Suárez & H.C. Evans, *Trichoderma pleuroticola* S.H. Yu & M.S. Park and *Trichoderma harzianum* Rifai—were obtained from the Applied Fungi Laboratory of Jilin Agricultural Science and Technology College for specificity testing. All strains were cultured on potato dextrose agar (PDA) at 25 °C in the dark. PDA was prepared by adding 20 g dextrose and 20 g agar to 1 L of potato decoction (obtained by boiling 200 g of potatoes per liter). Morphological characteristics of conidia and conidiophores were observed under an Olympus BX 53 microscope. Pathogenicity tests were performed following the method described by Fang [19]. All experiments were conducted in three independent biological replicates, each with three technical replicates.

To confirm the pathogenicity of strain XU0177, we followed the method described by Fang [19] with some specific details. A conidial suspension of the strain was prepared at 10^6^ spores/mL using sterile distilled water. Healthy *G. lingzhi* fruiting bodies at the primordium stage (about 2–3 cm in height) were surface sterilized with 75% ethanol. Each fruiting body was inoculated by spraying 1 mL of the conidial suspension onto the pileus and stipe; control fruiting bodies received the same volume of sterile water. Five fruiting bodies were used for each treatment. All inoculated fruiting bodies were incubated under standard cultivation conditions (25 °C, relative humidity >85%) and monitored daily for 14 days. For re-isolation, small pieces of tissue were cut from the margin of infected lesions, surface sterilized, and placed onto PDA medium. The resulting colonies were examined for morphological characteristics and further identified by RPB2 sequencing.

### 2.2. DNA Extraction and PCR

Genomic DNA of the target strain XU0177 and other *Trichoderma* isolates was extracted using a modified CTAB method [20]. Briefly, about 100 mg of fresh mycelium was ground in liquid nitrogen, then suspended in 600 µL of preheated CTAB buffer (2% CTAB, 1.4 M NaCl, 20 mM EDTA, 100 mM Tris-HCl, pH 8.0) and incubated at 65 °C for 60 min. After centrifugation, the supernatant was extracted with an equal volume of chloroform:isoamyl alcohol (24:1). DNA was precipitated with cold isopropanol, washed with 70% ethanol, air-dried, and dissolved in 50 µL of sterile ddH_2_O. The quality and concentration of DNA were assessed by 1% agarose gel electrophoresis and spectrophotometry.

The RPB2 fragment was amplified using primers fRPB2-5F (5′-GAYGAYMGWGATCAYTTYGG-3′) and bRPB2-7R2 (5′-ACYTGRTTRTGRTCNGGRAANGG-3′) [21]. PCR was performed in a 50 µL reaction mixture containing 25 µL of 2× Es Taq MasterMix (Dye), 1 µL each of forward and reverse primers (10 µmol/L), 10 µL of genomic DNA template, and 13 µL of sterile ddH_2_O. The thermal cycling program was as follows: initial denaturation at 94 °C for 5 min; 30 cycles of denaturation at 94 °C for 30 s, annealing at 55 °C for 30 s, and extension at 72 °C for 45 s; followed by a final extension at 72 °C for 10 min. PCR products were purified using a commercial kit (EasyPure Plasmid MiniPrep Kit, TransGen Biotech, Beijing, China) and sent to BGI (Beijing, China) for Sanger sequencing. All PCRs were performed in triplicate.

### 2.3. Sequences and Phylogenetic Analysis

The newly generated sequences were aligned with 21 sequences of RPB_2_ region retrieved from GenBank by using the Blastn algorithm. From GenBank, high-quality sequences of *Trichoderma* were retrieved and included in this study. *Hypocrea schweinitzii* was used as the outgroup. The ML analysis was performed using IQ-Tree v2.2.0 in Phylosuite v1.2.3, with the best model selected for each locus according to ModelFinder. The ML tree was evaluated by bootstrap analysis with 1000 replicates [22]. All the sequences used in this study are listed in Table 1.

### 2.4. Primers Design and Synthesis

The primers were designed using Primer Premier 5.0 based on the newly generated sequences obtained from strains XU0177:0177-2-1 (5′-CCGAGCTGGCCAACTATTTGAGACGT-3′) and 0177-2-2 (5′-TTCAAGCCGTTGGAGAGCGTGCC-3′). Primers were synthesized by BGI Co., Ltd., Beijing, China. The preliminary verification of primers specificity was in GeneBank (https://www.ncbi.nlm.nih.gov/).

### 2.5. Specificity and Sensitivity Test

Amplification of the RPB_2_ region using primers 0177-2-1 and 0177-2-2 was made for genomic DNA extracted from the five strains, with the procedure described before. The products of PCR amplification were resolved on a 1.0% agarose gel in JY 600 electrophoresis apparatus (Beijing JUNYI Electrophoresis Co., Ltd., Beijing, China). Images of the results were taken by a Canon 80D camera. Genomic DNA of strains XU0177 was diluted into 10^−1^ ng/µL, 10^−2^ ng/µL, 10^−3^ ng/µL, 10^−4^ ng/µL and 10^−5^ ng/µL, and 10 µL of each was used as a template for PCR amplification to test the sensitivity of newly designed primers. The experiment was repeated three times with consistent results.

### 2.6. Pathogens in Soil Test

Spore suspensions of 4.28 × 10^8^/mL were diluted into 4 concentrations and then mixed with the soil to produce soils with different spore contents: 4.28 × 10^6^/g, 4.28 × 10^5^/g, 4.28 × 10^4^/g, 4.28 × 10^3^/g and 4.28 × 10^2^/g [23]. The genomic DNA of soil microorganisms were extracted using an Ezup Column Soil DNA Purification Kit (Sangon Biotech Co., Ltd., Beijing, China). PCR amplification of the RPB_2_ region using primers 0177-2-1 and 0177-2-2 was performed and the products were resolved on a 1.0% agarose gel.

## 3. Results

### 3.1. Morphological Characteristics and Pathogenicity

**Figure 1:**
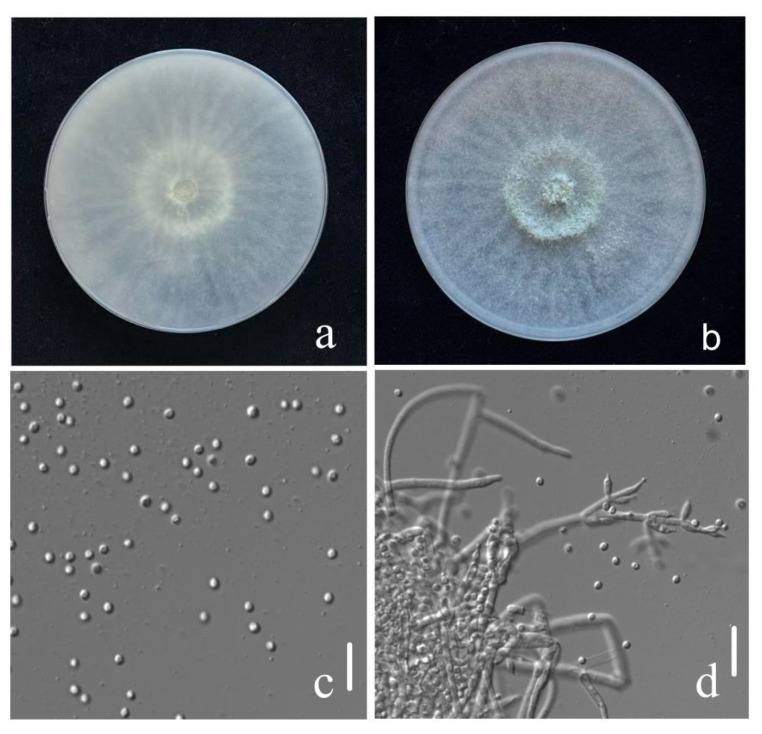
(**a**) Cultures on PDA (five days); (**b**) cultures on PDA (five days); (**c**) conidia; (**d**) conidiophores. Scale bars: 10 μm (**c**,**d**).

As shown in Figure 1, the colonies of the strain XU0177 are arranged in concentric rotation on the PDA medium under dark conditions of 25 °C; the initial colonies are green–gray and become black–green in the later period. Sporulating areas were black–green, occasionally with white patches. The colony reverse was initially colorless, later turning yellow due to the production of a yellow diffusible pigment, often accompanied by faint yellow striations. Conidia were ellipsoidal to narrowly ellipsoidal, measuring 3.2–5.3 × 2.2–3.5 μm. Based on these morphological characteristics and pathogenicity, the isolate was tentatively identified as *T. longibrachiatum*.

To confirm that strain XU0177 is the causal agent of green mold on *Ganoderma lingzhi*, a pathogenicity test was performed following the method of Fang. Healthy *G. lingzhi* fruiting bodies at the primordium stage were inoculated with a conidial suspension (10^6^ spores/mL) of strain XU0177, while control fruiting bodies received sterile distilled water. Each treatment consisted of five fruiting bodies. After five days of incubation under standard cultivation conditions (25 °C, high humidity), all five inoculated fruiting bodies developed typical green mold symptoms: gray-white mycelial growth on the surface, followed by green sporulation on the pileus and stipe. No symptoms appeared on any control fruiting bodies during the 14-day observation period. The fungus was re-isolated from the infected tissue margins onto PDA. The re-isolated colonies exhibited the same morphological characteristics as the original strain XU0177 (gray–green mycelium, concentric rings, and ellipsoidal conidia). The identity was further confirmed by RPB2 sequence analysis. Thus, Koch’s postulates were fulfilled, demonstrating that strain XU0177 is indeed the causal agent of green mold on *G. lingzhi*.

Based on the morphological characteristics and the pathogenicity test results combined with RPB2 phylogenetic analysis, the isolate was identified as *Trichoderma longibrachiatum*.

### 3.2. Phylogenetic Analysis

**Figure 2:**
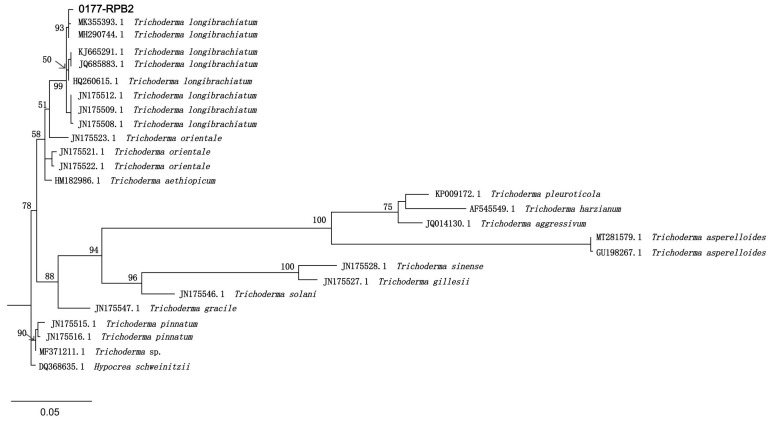
Maximum likelihood tree resulting from analysis of RPB2 sequence data with the outgroup *Hypocrea schweinitzii*.

The phylogenetic trees inferred from the RPB_2_ region showed that XU0177 is related to *T. longibrachiatum* with a high support rate (Figure 2). Combining with morphological characteristics identification, strain XU0177 is identified as *T. longibrachiatum*.

### 3.3. Specificity and Sensitivity of the PCR Assay and Test of Inoculated Soil

**Figure 3:**
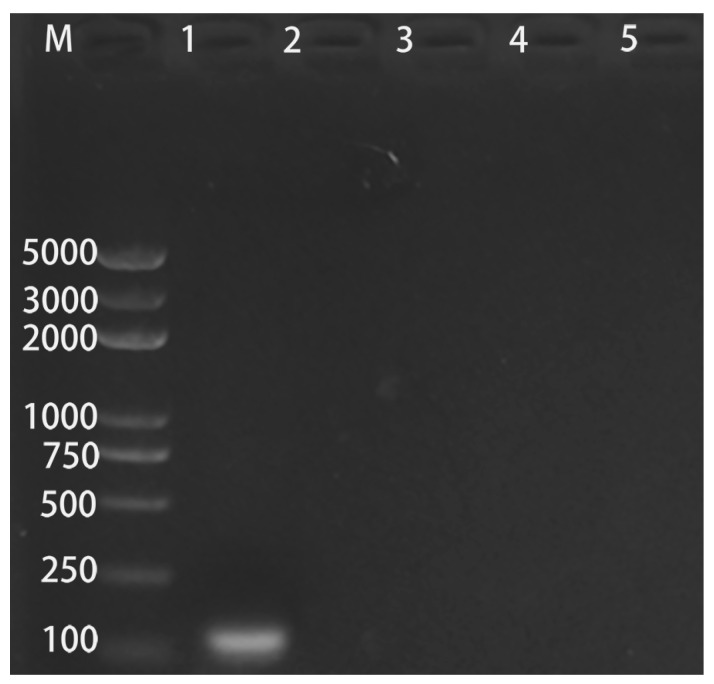
Gel electrophoresis results of specificity test. M: DNA marker; 1: Strain XU0177; 2: *T. pleuroticola*; 3: *T. koningiopsis*; 4: *T. atroviride*; 5: *T. harzianum*.

**Figure 4:**
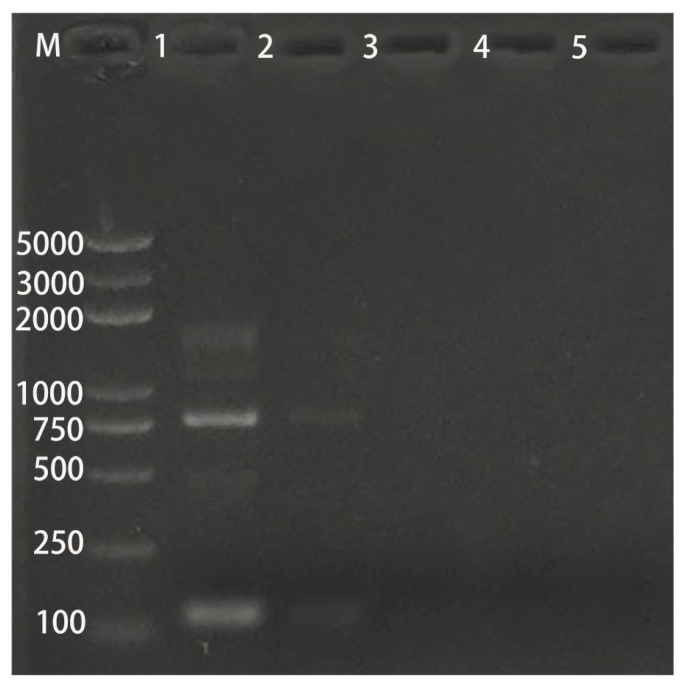
Gel electrophoresis results of sensitivity test. M: DNA marker; 1–5 represent template DNA with different concentrations: 10^−1^ ng/µL, 10^−2^ ng/µL, 10^−3^ ng/µL, 10^−4^ ng/µL, and 10^−5^ ng/µL.

**Figure 5:**
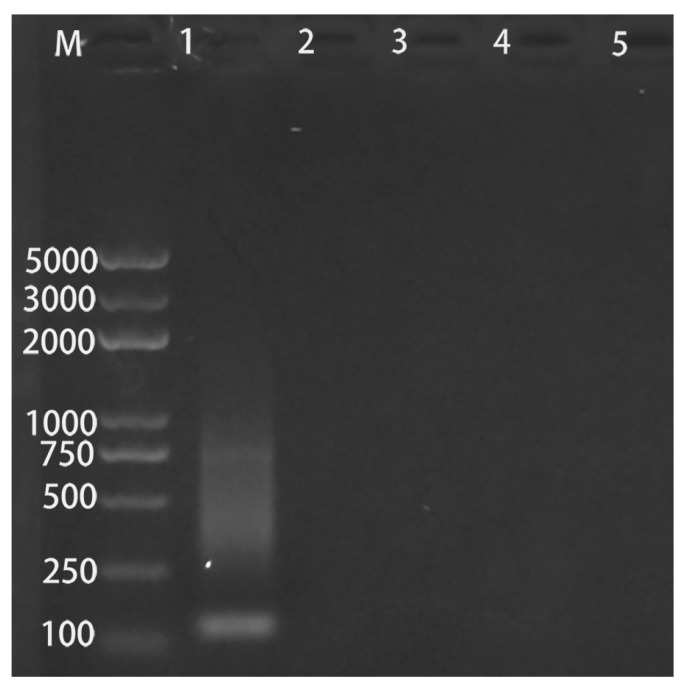
Gel electrophoresis results of PCR products in test of inoculated soil. M: DNA marker; 1–5 represent soil with different spore contents: 4.28 × 10^6^/g, 4.28 × 10^5^/g, 4.28 × 10^4^/g, 4.28 × 10^3^/g and 4.28 × 10^2^/g.

In the PCR amplification of the specificity verification, specific purpose bands were only present with the genomic DNA of the strain XU0177. No cross-reaction was observed with any of the non-target isolates, demonstrating that primers 0177-2-1 and 0177-2-2 are highly specific (Figure 3). The sensitivity of the assay was determined by testing serial ten-fold dilutions of XU0177 genomic DNA. The results of the sensitivity test show that the target band can still be visible after diluting the template DNA into 10^−1^ ng/µL, 10^−2^ ng/µL, and it can be informed that the minimum testing concentration of primers is 10^−2^ ng/µL (Figure 4). To assess the applicability of the method in a natural substrate, DNA was extracted from soil artificially inoculated with known amounts of *T. longibrachiatum* spores. Following PCR with primers 0177-2-1/0177-2-2, target amplicons were detected in soil samples containing 4.28 × 10^6^ spores per gram (Figure 5).

## 4. Conclusions

In summary, this study reports the first rapid and specific PCR-based detection system for *Trichoderma longibrachiatum* causing green mold on *Ganoderma lingzhi*. Here are the main novelties: (1) the assay was designed specifically for the unique growing conditions of *G. lingzhi*; (2) its detection limit is 10^−2^ ng/µL, which is ten times more sensitive than a previously reported PCR for a related Trichoderma species; (3) the primers are highly specific, showing no cross-reactivity with the four common Trichoderma pathogens we tested.

Of course, there are still some limitations that future studies should address. First, we only validated the assay with a limited number of isolates and artificially inoculated soil. It would be good to test it on a wider range of naturally infected field samples from different regions. Second, our method is qualitative and relies on conventional PCR equipment. Adapting it to a more field-friendly format—like LAMP or a lateral flow system—would make it easier to use in real-world settings.

Compared with previously reported molecular assays for other *Trichoderma* species, our system shows competitive performance. For example, a conventional PCR assay for *T. pleuroticola* achieved a detection limit of 0.1 ng/µL (Huang, 2018 [6]), while our assay reaches 0.01 ng/µL for *T. longibrachiatum*. A qPCR method for *T. harzianum* reported a limit of 10 pg/µL but requires expensive equipment and reagents. LAMP assays for *T. atroviride* are rapid but prone to cross-contamination. In terms of specificity, our primers showed no cross-reactivity with four closely related *Trichoderma* species (*T. atroviride*, *T. koningiopsis*, *T. pleuroticola*, and *T. harzianum*), which is comparable to or better than most published PCR-based methods. According to the analyses of the RPB_2_ sequence, the designed primers 0177-2-1 and 0177-2-2 were specific to the target strain, and showed no amplification with other similar species. Therefore, this detection system can be used for the specific detection of *T. longibrachiatum*, which could recognize the difference with other species.

Despite these limitations, our work provides a practical tool for early diagnosis and disease monitoring in *G. lingzhi* production. The primer set can also serve as a useful reference for developing similar detection assays for other Trichoderma pathogens in edible mushroom cultivation.

## 5. Discussion

In addition to the experimental limitations discussed above, there is another theoretical point worth discussing, which concerns the genetic similarity between *T. longibrachiatum* and some clinically relevant strains. As shown by Hatvani et al. [24], *T. longibrachiatum* strains from agricultural sources are genetically very close to those causing human infections, and even to the closely related species *T. bissettii*. In fact, those authors proposed reclassifying *T. bissettii* as *T. longibrachiatum* f. sp. bissettii because traditional genetic markers, including RPB2, often fail to distinguish them. So, could our primers amplify DNA from these clinically relevant strains or from *T. bissettii* and cause false positives?

We think that the answer is no, and here is why. First, our assay was never meant to be a universal taxonomic tool for clinical diagnosis. It is a practical detection method for a very specific problem: green mold caused by *T. longibrachiatum* in *G. lingzhi* cultivation. The identity of our target strain XU0177 was confirmed by a combination of morphology, pathogenicity tests on *G. lingzhi*, and RPB2 phylogeny, which is sufficient for an agricultural pathogen.

Second, and more importantly, *T. bissettii* has never been reported as a pathogen causing green mold on any Ganoderma species. To date, no study has reported *T. bissettii* as a pathogen of Ganoderma or any cultivated mushroom, nor have clinical *T. longibrachiatum* strains been found in mushroom growing substrates. The same goes for clinical strains of *T. longibrachiatum*; they are unlikely to appear in mushroom growing substrates. So, even if our primers could theoretically amplify those targets in a pure culture test, the chance of actually encountering them in a *G. lingzhi* farm is extremely low.

Given that the target environment of our assay (mushroom cultivation substrates) does not harbor these clinically relevant strains or *T. bissettii*, the theoretical cross-reactivity has no practical consequence. Therefore, while we fully acknowledge the taxonomic complexity highlighted by Hatvani et al. [24], we believe that it does not compromise the practical utility of our assay for its intended agricultural application.

## Figures and Tables

**Table 1:** Sequences used in phylogenetic analysis.

Species	Isolate/Strain	GenBank Accession Number
		RPB_2_
*Trichoderma longibrachiatum*	S328	KJ665291.1
*Trichoderma longibrachiatum*	S328	JQ685883.1
*Trichoderma longibrachiatum*	ATCC 18648	HQ260615.1
*Trichoderma longibrachiatum*	LS0017	MH290744.1
*Trichoderma longibrachiatum*	GJS 08-198	JN175508.1
*Trichoderma longibrachiatum*	GJS 04-53	JN175512.1
*Trichoderma longibrachiatum*	GJS 04-31	JN175509.1
*Trichoderma longibrachiatum*	RT3	MK355393.1
*Trichoderma aethiopicum*	C.P.K. 1837	HM182986.1
*Trichoderma orientale*	DIS 270F	JN175521.1
*Trichoderma orientale*	GJS 09-784	JN175522.1
*Trichoderma pleuroticola*	TRS70	KP009172.1
*Trichoderma harzianum*	CBS 226.95	AF545549.1
*Trichoderma aggressivum*	CBS100525	JQ014130.1
*Trichoderma asperelloides*	T145	MT281579.1
*Trichoderma sinense*	DAOM 230004	JN175528.1
*Trichoderma asperelloides*	GJS 99-9	GU198267.1
*Trichoderma gracile*	GJS 10-263	JN175547.1
*Trichoderma solani*	GJS 08-81	JN175546.1
*Trichoderma gillesii*	GJS 00-72	JN175527.1
*Hypocrea schweinitzii*	GJS 01-364	DQ368635.1
*Trichoderma* sp.	TC714	MF371211.1
*Trichoderma pinnatum*	GJS 04-100	JN175515.1
*Trichoderma pinnatum*	GJS 02-120	JN175516.1
*Trichoderma orientale*	GJS 10-230	JN175523.1

## Data Availability

The original contributions presented in this study are included in the article. Further inquiries can be directed to the corresponding author.
